# Comparative effects of dietary stigmasterol and oxidised stigmasterol on cholesterol absorption and metabolism in mice

**DOI:** 10.1017/S0007114525105357

**Published:** 2025-12-14

**Authors:** Yui Ohara, Kyoichi Osada

**Affiliations:** Department of Agricultural Chemistry, School of Agriculture, Meiji University Graduate School, 1-1-1 Higashimita, Tama-ku, Kawasaki, Kanagawa 214-8571, Japan

**Keywords:** Stigmasterol, Oxidised stigmasterol, Phytosterols, Oxidised phytosterols, Cholesterol metabolism, Mice

## Abstract

Dietary phytosterols exert hypocholesterolemic effects by inhibiting cholesterol absorption in the small intestine. However, oxidised phytosterols exert harmful effects. In this study, we compared the effects of dietary stigmasterol or oxidised stigmasterol (OS) on cholesterol absorption and metabolism in mice. Institute of Cancer Research (ICR) male mice were fed one of the following diets: a standard American Institute of Nutrition (AIN) diet; the standard diet plus 0·25 % cholesterol; the standard diet plus 0·25 % cholesterol and 0·25 % stigmasterol or the standard diet plus 0·25 % cholesterol and 0·25 % OS. Stigmasterol, but not OS, decreased plasma total cholesterol levels. Unlike stigmasterol, dietary OS increased the cholesterol levels in micellar solutions. Thus, OS could not exert hypocholesterolemic effects as it could not displace cholesterol in micellar solutions. In contrast, dietary OS downregulates the mRNA expression of genes involved in cholesterol synthesis and upregulates the mRNA expression of genes involved in cholesterol catabolism in mice fed cholesterol. In addition, dietary stigmasterol and OS increased the levels of faecal-neutral steroids by downregulating the mRNA expression of Niemann-Pick C1-like 1 protein (NPC1L1) in the small intestine. Dietary stigmasterol may directly regulate the mRNA expression of NPC1L1, whereas dietary OS may reduce the mRNA expression of sterol regulatory element-binding protein 2 and act as a Liver X receptor *α* agonist, reducing the mRNA expression of NPC1L1. Therefore, OS may affect cholesterol absorption and metabolism through a mechanism different from that of stigmasterol.

## Graphical abstract

**Figure:**
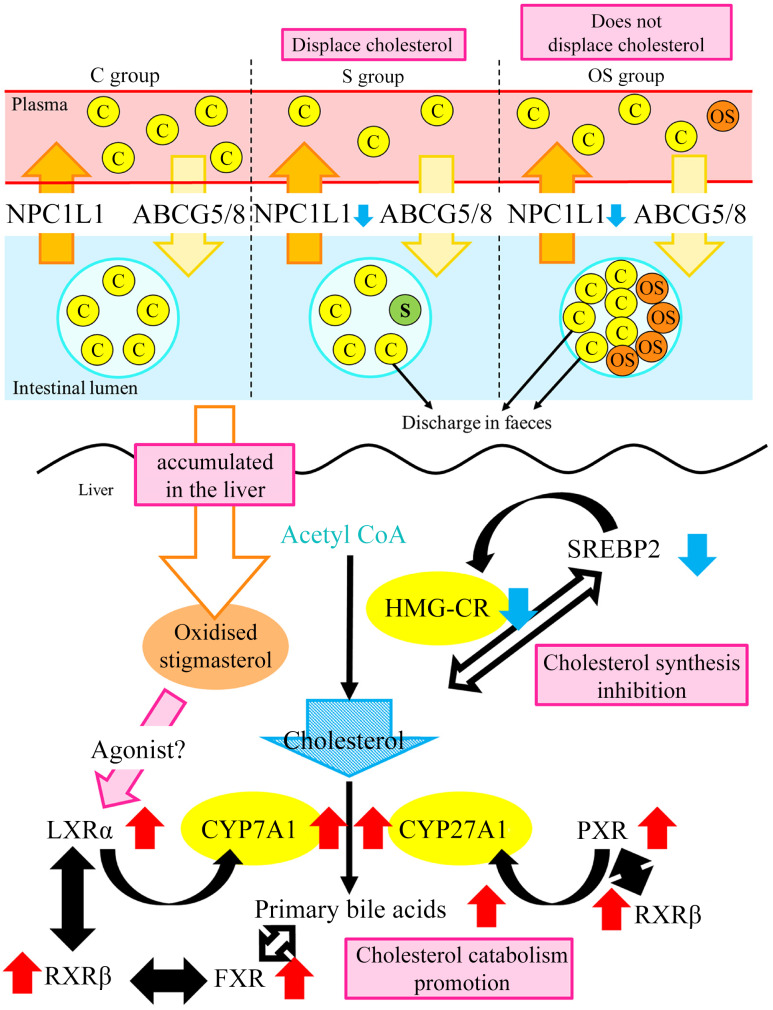

The intake levels of phytosterols in Japan, North America and Spain are approximately 370^(1)^, 360^(2)^ and 280^(3)^ mg/d, respectively, which are similar to that of cholesterol^(1)^. Because dietary phytosterols exert hypocholesterolemic effects^(4)^, functional foods and supplements enriched with phytosterols are expected to increase in the future.

Phytosterols, similar to cholesterol, can be oxidised to form various derivatives. Because oxidised phytosterols are generated during the heating of vegetable oils, significant amounts of oxidised phytosterols are particularly found in fried foods such as potato chips^(5)^ and French fries^(6)^. Moreover, oxidised phytosterol levels in food have been reported to increase upon microwave heating^(7)^. Scholz *et al.* ^(8)^ reported that oxidised phytosterols comprise 1·9 % of the daily phytosterols intake and estimated that the daily intake of oxidised phytosterols ranges from 1·2 to 2·9 mg/d for nonheated foods, 3·5 to 4·2 mg/d for heated milk and 29·6 mg/d for liquid spread for cooking and baking.

Oxidised phytosterols exert various harmful effects, such as cytotoxicity^(9)^, apoptosis induction^(9)^, inflammation^(10)^ and promotion of atherosclerosis^(11)^, similar to oxidised cholesterol^(12)^. Liang *et al.* ^(13)^ found that dietary oxidised phytosterols inhibit cholesterol metabolism *in vivo*. Laparra *et al.* ^(14)^ revealed that oxidised phytosterol affects cholesterol metabolism by downregulating the mRNA expression of 3-hydroxy-3-methylglutaryl-coenzyme A reductase (HMG-CR) and sterol regulatory element-binding protein 2 (SREBP2), which are involved in cholesterol synthesis, and ATP-binding cassette subfamily G member 5 (ABCG5), which is involved in sterol excretion *in vivo*. Moreover, Koyama *et al.* ^(15)^ reported that dietary oxidised phytosterols change fatty acid composition and increase the proportion of arachidonic acid by upregulating the mRNA expression of fatty acid desaturase in the liver. Thus, multiple studies have examined the effects of oxidised phytosterols on cholesterol metabolism; however, the specific effects of individual oxidised phytosterols remain unclear.

Stigmasterol is a major phytosterol, and its structure differs from cholesterol, campesterol and *β*-sitosterol by a double bond at the C22–23 position^(16)^. The bioavailability of stigmasterol is significantly lower than that of campesterol or *β*-sitosterol^(17)^. Some studies have shown that stigmasterol exerts useful pharmacological effects, including anti-inflammatory^(18)^, anti-diabetic^(19)^ and anti-cancer activities^(20)^. Therefore, stigmasterol may be used in healthy foods and medicines in the future. Multiple studies have suggested that oxidised phytosterols, in contrast to unoxidised phytosterols, are readily absorbed by the small intestine^(21)^. Thus, once absorbed, oxidised phytosterols may exert harmful effects similar to oxidised cholesterol.

Previously, we reported that dietary oxidised stigmasterol (OS) accumulates in the liver and then induces the production of reactive oxygen species and modulates the antioxidant system in mice^(22)^. In this study, we aimed to study the different effects of dietary stigmasterol or OS on cholesterol metabolism, particularly their potential harmful effects in mice. At present, the effect of oxidised phytosterols, particularly that of the individual oxidised phytosterols, on cholesterol metabolism has been scarcely studied *in vivo*. Therefore, this study constitutes one of the first in mice to examine the effects of dietary OS on cholesterol metabolism.

## Methods

### Preparation of purified oxidised stigmasterol

Stigmasterol (Biosynth, Ltd.) was heated at 170℃ for 6 h. The heated stigmasterol was dissolved in diethyl ether and applied to a silica gel column (Silica Gel 60, Spherical; Nacalai Tesque, Inc.). The unoxidised and decomposed stigmasterol was eluted with diethyl ether, and the fraction containing OS was eluted with methanol. The fraction eluted with methanol was re-dissolved in hexane and diethyl ether (1:1, v/v) after the solvent was removed and then reapplied to a silica gel column. Subsequently, hexane and diethyl ether (1:1, v/v), followed by diethyl ether, were poured into the column to remove the unoxidised stigmasterol. Finally, purified OS was eluted with methanol and dried using a rotary evaporator.

### Analysis of oxidised stigmasterol

A portion of the purified OS was converted to trimethylsilyl ether in a mixture of trimethylchlorosilane1,1,1,3,3,3-hexamethyldisilazane, and dried pyridine (1:3:9, v/v/v) for 30 min at 60℃ after 5*α*-cholestane was added to an aliquot as an internal standard. OS was identified using GC–MS. GC–MS analyses were performed on Shimadzu GC-2010 and Shimadzu PARVUM2 instruments (Shimadzu Co.) with a DB-5MS column (0·25 mm, 60·0 m × 0·25 μm I.D.; Agilent Technologies Ltd.). Helium was used as the carrier gas at a flow rate of 1·08 ml/min and a split ratio of 1:10. The column oven, evaporation chamber, ion source and interface temperatures were set at 270, 300, 200 and 300℃, respectively. The electron ionisation mode mass spectrum was obtained at 70 eV and measured within a mass range of 35–1000 m/z.

Major OS derivatives were identified based on retention time and characteristic m/z, as reported by Menéndez-C *et al.* ^(23)^, Conchill *et al.* ^(24)^, Leal-C *et al.* ^(25)^ and Foley *et al.* ^(16)^. The prepared OS consisted of the following components: 0·2 % unoxidised stigmasterol; 5·8 % stigmasta-5,22-dien-3*β*,4*β*-diol (4*β*-hydroxystigmasterol); 4·8 % stigmasta-5,22-diene-3*β*,7*α*-diol (7*α*-hydroxystigmasterol); 11·0 % stigmasta-5,22-diene-3*β*,7*β*-diol (7*β*-hydroxystigmasterol); 10·2 % 5*α*,6*α*-epoxystigmast-22-en-3*β*-ol (5*α*,6*α*-epoxystigmasterol); 10·1 % 5*β*,6*β*-epoxystigmast-22-en-3*β*-ol (5*β*,6*β*-epoxystigmasterol); 6·2 % stigmastanetriol (stigmatriol); 22·3 % stigmasta-5,22-dien-7-on-3*β*-ol (7-ketostigmasterol) and 29·4 % unidentified OS. Unidentified OS was composed of seventeen unknown peaks.

### Animals and diet

The animal experiments were conducted according to the guidelines of the Ethics Committee of Experimental Animal Care at Meiji University (Kanagawa, Japan) (approval code: MUIACUC2020-01).

ICR mice (6-week-old healthy males with no genetic modifications and previous procedures; Japan SLC, Inc.) were housed individually in a temperature (24℃) and light-controlled (07.00–19.00) windowless room, and animal cage locations were not rotated during the experiment. All treatments were performed by the same experimenter. After eight days of acclimation, 22 ICR mice were divided randomly into four groups to avoid differences in the initial body weight: the standard (St) group (five mice) was fed an AIN standard diet, the control (C) group (five mice) was fed a standard diet supplemented with 0·25 % cholesterol, the stigmasterol (S) group (six mice) was fed a standard diet supplemented with 0·25 % cholesterol and 0·25 % stigmasterol and OS group (six mice) fed a standard diet supplemented with 0·25 % cholesterol and 0·25 % OS. The diets were prepared according to the AIN76 recommendations^(26)^, and the detailed composition of the diets is shown in Table 1. The mice in each group were fed the same quantity (in grams) of their respective diets to avoid differences in intake levels among the four groups. The feeding period was set at 14 d to ensure consistency with our previous study^(22)^. After 14 d, blood was collected from the mice via cardiac puncture after anaesthetisation with isoflurane using a syringe containing heparin Na (Mochida Pharmaceutical Co., Ltd.) as an anticoagulant by alternating sequence among groups (St1→C1→S1→OS1→St2→…). The liver and mucosa of the small intestine were immediately excised, and the plasma was separated by centrifugation at 3000 rpm for 25 min. These tissues were kept at –80℃ until further analyses. Some liver and mucosa of the small intestine samples were used for RNA analysis after immersion in RNAlater solution (Thermo Fisher Scientific Inc.) at 4℃ for 1 d. Faecal samples were collected for 4 days (beginning four days before euthanasia) and lyophilised. Blinding was not performed. All stages of the experiment, including group allocation, conduct, outcome assessment and data analysis, were carried out by a single experimenter who was aware of group assignments.

**Table 1.** Diet composition

Component	Group			
	St	C	S	OS
	(%)			
Corn starch^*^	15	15	15	15
Casein^†^	20	20	20	20
Sucrose^‡^	48·43	48·18	47·93	47·93
High linoleic safflower oil^§^	5	5	5	5
Cellulose^†^	5	5	5	5
Mineral Mix. (AIN76)^||^	5	5	5	5
Vitamin Mix. (AIN76)^||^	1	1	1	1
Choline bitarttrate^¶^	0·2	0·2	0·2	0·2
DL-Methionine^¶^	0·3	0·3	0·3	0·3
Cholesterol^¶^	0·00	0·25	0·25	0·25
Stigmasterol^**^	0·00	0·00	0·25	0·00
Oxidised stigmasterol	0·00	0·00	0·00	0·25
Sodium cholate^¶^	0·075	0·075	0·075	0·075

St: standard diet fed group; C: 0·25 % cholesterol fed group; S: 0·25 % cholesterol and 0·25 % stigmasterol fed group; OS: 0·25 % cholesterol and 0·25 % oxidised stigmasterol fed group.

* Nihon Shokuhin Kako Co., Ltd.

† Feed One Co., Ltd.

‡ Mitsui. DM Sugar Co., Ltd.

§ Nisshin OilliO Group, Ltd.

|| Oriental Yeast Co., Ltd.

¶ Nacalai Tesque, Inc.

** Biosynth Ltd.

### Analyses of plasma and hepatic total cholesterol levels

Plasma total cholesterol (TC) levels were measured using commercial kits (Fujifilm Wako Pure Chemical Industries Ltd.). Hepatic lipids were extracted as described by Folch *et al.* ^(27)^. Hepatic TC levels were measured as described by Sperry *et al.* ^(28)^.

### Analyses of the faecal steroid levels

Neutral and acidic faecal steroids were extracted as described by Isaksson *et al.* ^(29)^. Neutral steroids were analysed using a gas chromatograph GC-2025 (Shimadzu Co.) equipped with an FID and a ZB-5MS column (0·25 mm, 60·0 m × 0·25 um I.D.; Phenomenex), with 5*α*-cholestane as the internal standard, as described by Sugano *et al.* ^(30)^. Nitrogen was used as the carrier gas at a flow rate of 1·0 ml/min. The column oven and injector temperatures were set at 240 and 300°C, respectively.

Acidic steroids were analysed using a gas chromatograph GC-2014 (Shimadzu Co.) equipped with an FID and a TC-5 column (0·25 mm, 30·0 m × 0·25 um I.D.; GL Sciences), with 23-nor-deoxycholic acid as the internal standard, as described by Sugano *et al.* ^(30)^. Nitrogen was used as a carrier gas at a flow rate of 1·5 ml/min. The column oven and injector temperatures were set at 230–270°C (2°C/min temperature elevation) and 310°C, respectively.

### RNA extraction from liver and mucosa of the small intestine

Total RNA was extracted from liver and small intestinal mucosal tissues using Sepasol-RNA I Super G (Nacalai Tesque, Inc.). It was then mixed with a 6 M lithium chloride solution for RNA purification. The RNA concentration was measured using a NanoDrop Lite spectrophotometer (Thermo Fisher Scientific Inc.).

### Oligonucleotide primer sequences

The primers used to detect mouse *β*-actin (Gene ID: 11461), sterol regulatory element-binding protein 2 (SREBP2; Gene ID: 20788), 3-hydroxy-3-methylglutaryl-coenzyme A reductase (HMG-CR; Gene ID: 15357), liver X receptor *α* (LXR*α*: NR1H3; Gene ID: 22259), pregnane X receptor (PXR: NR1I2; Gene ID: 18171), Farnesoid X receptor (FXR: NR1H4; Gene ID: 20186), retinoid X receptor *β* (RXR*β*: NR2B2; Gene ID: 20182), cytochrome P450 family 7 subfamily A member 1 (CYP7A1: cholesterol 7*α*-hydroxylase; Gene ID: 13122), cytochrome P450 family 27 subfamily A member 1 (CYP27A1: sterol 27-hydroxylase; Gene ID: 104086), Niemann-Pick C1-like 1 protein (NPC1L1; Gene ID: 237636), ATP binding cassette subfamily G member 5 (ABCG5; Gene ID: 27409) and ATP binding cassette subfamily G member 8 (ABCG8; Gene ID: 67470) via reverse transcription PCR were designed using Primer3Plus software (https://www.bioinformatics.nl/cgi-bin/primer3plus/primer3plus.cgi). Primers synthesised by Eurofins Genomics (Tokyo, Japan) were designed to flank known or putative introns of the target gene, thereby preventing amplification of contaminating genomic DNA. The primer sequences were as follows: *β*-actin, 5′-TGTCGAGTCGCGTCCACC-3′ (sense) and 5′-TATCGTCATCCATGGCGAACTGG-3′ (antisense); SREBP2, 5′-CAGGGAACTCTCCCACTTGA-3′ (sense) and 5′-GAGACCATGGAGACCCTCAC-3′ (antisense); HMG-CR, 5′-CCAGGATGCAGCACAGAATGT-3′ (sense) and 5′-CCAATTC GGGCAAGCTGCCG-3′ (antisense); LXR*α*, 5′-TGCCATCAGCAT CTTCTCTG-3′ (sense) and 5′-GGCTCACCAGCTTCATTAGC-3′ (antisense); PXR, 5′-GACCTGCCTATTGAGGACCA-3′ (sense) and 5′-TTCTGGAAGCCACCATTAGG-3′ (antisense); FXR, 5′-TA CCACTACAACGCGCTCAC-3′ (sense) and 5′-ACATCCCCAT CTCTCTGCAC-3′ (antisense); CYP7A1, 5′-GAGCCCTGAAGC AATGAAAG-3′ (sense) and 5′-GCTGTCCGGATATTCAAGGA-3′ (antisense); RXR*β*, 5′-CTTGGTCTCCAAGTCGAAGG-3′ (sense) and 5′-GCCAAATGAGAAGGAAGCAG-3′ (antisense); CYP27A1, 5′-CACTTTCCTTCCCAAATGGA-3′ (sense) and 5′-AGGAAGT GCAGGTAGCCAGA-3′ (antisense); NPC1L1, 5′-GGAAATGCA ATCCTTCCAGA-3′ (sense) and 5′-GCCAGGGAGATGTACAG GAA-3′ (antisense); ABCG5, 5′-TCACTTGCATTTGCTTCCTG-3′ (sense) and 5′-TTGCTGACGCTGTAGGACAC-3′ (antisense) and ABCG8, 5′-TCTCCAGGTCCTGATTGGTC-3′ (sense) and 5′-GGGTGCTTTTGACTCTGCTC-3′ (antisense).

### Real-time quantitative PCR

One microgram of RNA was incubated at 65℃ for 5 min and then quickly cooled on ice. Reverse transcription of RNA was performed using a ReverTra Ace qPCR RT Master Mix (Toyobo Co., Ltd.) with the temperature increased to 37℃ for 15 min, followed by 98℃ for 5 min. An aliquot of the generated cDNA samples was mixed with 9 µl THUNDERBIRD NEXT SYBR qPCR Mix (Toyobo Co., Ltd.) in the presence of 12 nmol each of the sense and antisense primers for *β*-actin and the target gene. This reaction mix was then subjected to the following cycling conditions in a Thermal Cycler Dice Real Time System III (Takara Bio Inc.): one cycle at 95℃ for 1 min. The results (fold changes) are expressed as relative fold changes based on comparisons with the amount of RNA of the target gene to that of *β*-actin (the internal control) using the equation, 2^−Cq(target)+Cq(*β*-actin)^.

### Effect of stigmasterol or oxidised stigmasterol on the incorporation of cholesterol into micellar solutions

A mixed micellar solutions composed of 40 ml 15 mM sodium phosphate buffer (pH 7·4) containing 308·6 mg NaCl (Nacalai Tesque, Inc.), 16·4 mg phosphatidylcholine (from Egg Yolk, Nacalai Tesque, Inc.), 127·8 mg sodium taurocholate, 40 mg trilinolenin (Olbracht Serdary Research Laboratories), 35 mg cholesterol (Nacalai Tesque, Inc.) (C group) and 35 mg stigmasterol (S group) or OS group (four samples each) were prepared by sonication as described by Ogino *et al.* ^(31)^ The prepared micellar solutions were incubated at 37°C for 1 h and then centrifuged at 1000 g for 20 min. The supernatant was filtered using a 220 nm filter (Membrane Solutions LLC). Lipids were extracted from the filtered solution as described by Folch *et al.* ^(27)^. Thereafter, sterol levels were analysed by GC–MS, as described in the previous section.

### Statistical analyses and justification of sample size

Based on studies performed in rats and mice administered cholesterol, cholesterol + phytosterols or cholesterol + oxidised phytosterols, with group sizes of 7–12 rodents per group^(20)^, this study was designed with five or six mice per experimental group, consistent with the group sizes used in our previous study^(22)^. No prior inclusion or exclusion criteria were specified. All animals and data points were included in the experiment and analysis. All data are expressed as the mean (standard error of the mean (sem)). Statistical analyses were performed using Excel Statistics version 4.08 (Social Survey Research Information Co., Ltd.). The Shapiro–Wilk test was used to evaluate normality, revealing that the majority of variables, except some growth parameters, acidic steroid levels and mRNA expression levels in the mucosa of the small intestine, were normally distributed. Therefore, if the data were normally distributed, the Student’s *t* test was used to evaluate significant differences between values of the St and C groups, and one-way ANOVA and the Tukey–Kramer test were used to evaluate significant differences among values of the C, S and OS groups. If the data were not normally distributed, the exact Mann–Whitney *U* test was used to evaluate significant differences between the St and C groups, and the Kruskal–Wallis and Steel-Dwass tests were used to evaluate significant differences among the C, S and OS groups. Statistical significance was set at *P* < 0·05. No data on the effect size were available for power analysis because this was an exploratory study comparing the effects of dietary stigmasterol or OS on cholesterol metabolism in mice. Instead of conducting a conventional power analysis, minimally detectable effect sizes were estimated using G * Power version 3.1.9.7 based on predetermined sample sizes. For the St and C group comparisons (*n* 5 each), the minimally detectable effect size (Cohen’s d) was approximately 2·02, assuming a two-sided significance level of 0·05, and a statistical power of 80 %. For the C, S and OS groups (*n* 5, 6 and 6, respectively) comparison, the minimally detectable effect size (Cohen’s f) for the one-way ANOVA was approximately 0·84 under the same assumptions.

## Results

### Effects of dietary stigmasterol or oxidised stigmasterol on growth parameters

Liver weight tended to be slightly higher in the C group than in the St group (*P* = 0·06) (Table 2). However, no significant effects were observed on the other growth parameters among the four groups.

**Table 2.** Effect of dietary stigmasterol or oxidised stigmasterol on growth parameters

	Group							
	St		C		S		OS	
	Mean	sem	Mean	sem	Mean	sem	Mean	sem
	(g/d)							
Food intake	5·4	0·0	5·4	0·0	5·5	0·0	5·4	0·0
	(g)							
Initial body weight	36·3	1·7	36·3	0·6	36·3	0·4	36·3	0·4
Final body weight	39·9	0·7	39·7	0·8	39·2	1·0	39·8	0·5
Weight gain	3·6	1·1	3·5	0·6	2·9	1·0	3·5	0·7
	(g/10 g body weight)							
Liver weight	0·49	0·03	0·59	0·04	0·53	0·01	0·56	0·02
Visceral WAT weight	0·58	0·04	0·57	0·03	0·56	0·01	0·58	0·06
Subcutaneous WAT weight	0·26	0·04	0·20	0·02	0·20	0·02	0·23	0·03
Total WAT weight	0·83	0·07	0·78	0·05	0·77	0·03	0·80	0·08

Data are presented as the mean and sem of 5–6 mice in each group. WAT: white adipose tissue. Other abbreviations are the same as shown in Table 1.

### Effect of dietary stigmasterol or oxidised stigmasterol on plasma and hepatic cholesterol levels

**Figure 1.**
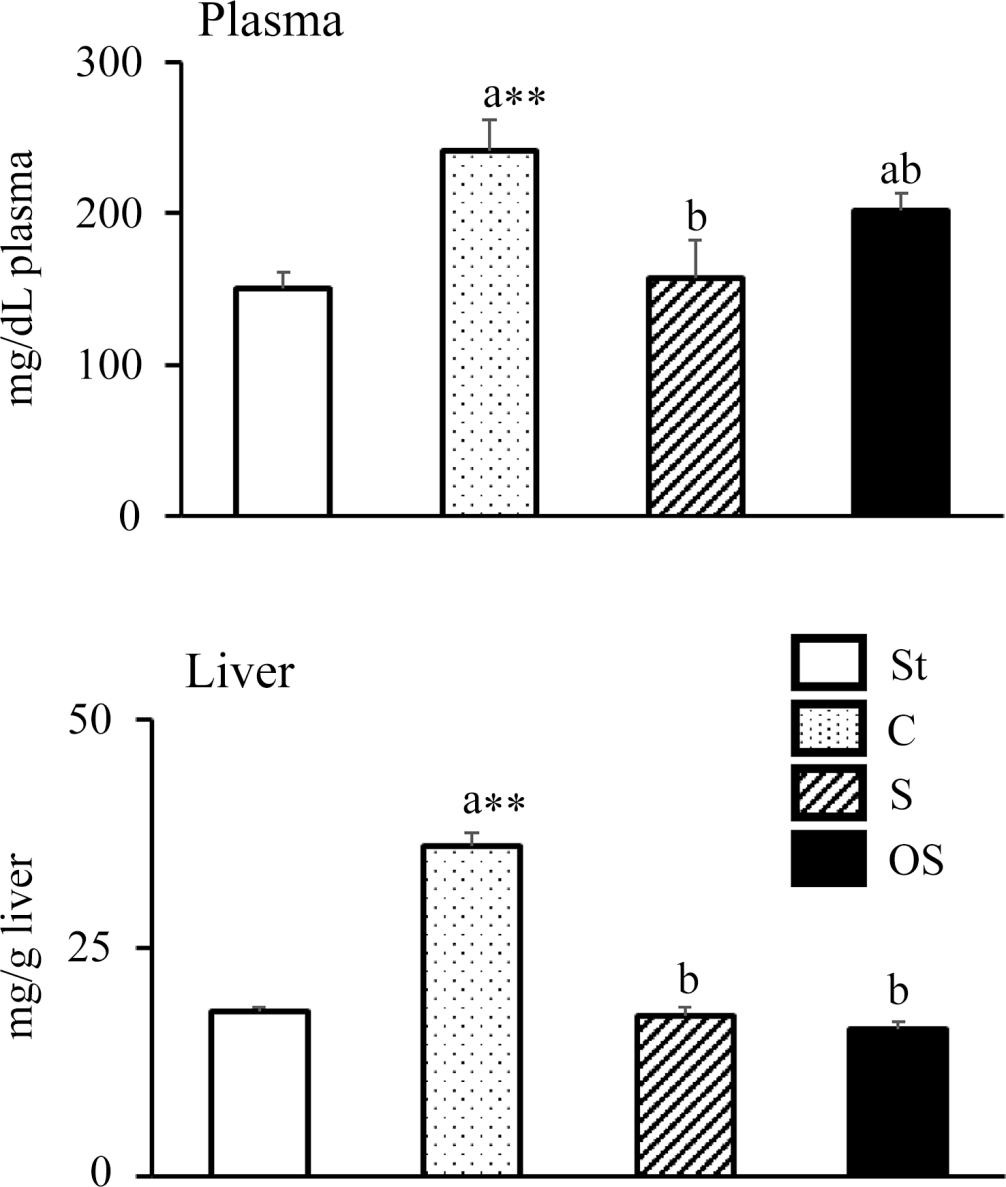
Effects of dietary stigmasterol or oxidised stigmasterol on plasma and hepatic cholesterol levels. Data are presented as the mean and sem of 5–6 mice in each group. Significant differences between values of the St and C groups at *P* < 0·01**. ^ab^Values without a common superscript letter are significantly different among values of the C, S and OS groups at *P* < 0·05. Other abbreviations are the same as shown in Table 1. Plasma cholesterol level was the level per dl of plasma, and hepatic cholesterol level was the level per gram of tissue.

Plasma TC levels were significantly higher in the C group than in the St group, but were significantly lower in the S group than in the C group (Figure 1). However, no significant effect was observed in the OS group compared with the C and S groups. Hepatic TC levels were significantly higher in the C group than in the St group, but were significantly lower in the S and OS groups than in the C group.

### Effect of dietary stigmasterol or oxidised stigmasterol on the faecal steroid levels

The faecal cholesterol and neutral steroid levels were significantly higher in the C group than in the St group and significantly higher in the S and OS groups than in the C group (Table 3). The coprostanol levels were significantly higher in the C group than in the St group and significantly higher in the OS group than in the C and S groups.

**Table 3.** Effects of dietary stigmasterol or oxidised stigmasterol on faecal neutral steroid levels

Component	Group							
	St		C		S		OS	
	Mean	sem	Mean	sem	Mean	sem	Mean	sem
	(mg/d)							
Cholesterol	0·85	0·10	6·47	1·71^a*^	14·45	2·07^b^	15·49	2·27^b^
Coprostanol	0·03	0·00	0·10	0·03^a*^	0·07	0·01^a^	0·29	0·05^b^
Total neutral steroids	0·88	0·10	6·57	1·73^a*^	14·52	2·09^b^	15·78	2·26^b^

Data are presented as the mean and sem of 5–6 mice in each group. Significant differences between values of the St and C groups at *P* < 0·05*. ^ab^Values without a common superscript letter are significantly different among values of the C, S and OS groups at *P* < 0·05. Other abbreviations are the same as shown in Table 1. Total neutral sterolids represent the sum of cholesterol and coprostanol.

The faecal cholic acid levels did not differ between the St and C groups, the C and S groups and the C and OS groups, but were significantly lower in the OS group than in the S group (Table 4). The total levels of *α*-muricholic acid and chenodeoxycholic acid were significantly higher in the C group than in the St group, but did not differ among the C, S and OS groups. The *β*-muricholic acid level was significantly higher in the C group than in the St group and significantly higher in the OS group than in the C and S groups. Total primary bile acid levels were significantly higher in the C group than in the St group and significantly higher in the OS group than in the C and S groups. The total levels of ω-muricholic acid and deoxycholic were significantly higher in the C group than in the St group, but significantly lower in the S group than in the C group. The lithocholic acid level tended to be slightly higher in the C group than in the St group (*P* = 0·10), but was significantly lower in the S group than in the C group. The hyodeoxycholic acid level did not differ between the St and C groups, the C and S groups or the C and OS groups, but tended to be slightly lower in the OS group than in the S group (*P* = 0·06). The ursodeoxycholic acid levels tended to be slightly higher in the C group than in the St group (*P* = 0·05) and were significantly higher in the S and OS groups than in the C group. The total secondary bile acids and total bile acids levels were significantly higher in the C group than in the St group, but did not differ among the C, S and OS groups.

**Table 4.** Effects of dietary stigmasterol or oxidised stigmasterol on faecal acidic steroid levels

Component	Group							
	St		C		S		OS	
	Mean	sem	Mean	sem	Mean	sem	Mean	sem
	(mg/d)							
Cholic acid	0·29	0·03	0·19	0·07^ab^	0·23	0·01^a^	0·08	0·03^b^
*α*-Muricholic aicd + Chenodeoxycholic acid	0·01	0·01	0·08	0·01**	0·06	0·01	0·08	0·03
*β*-Muricholic acid	0·35	0·10	0·75	0·10^a*^	0·62	0·07^a^	1·09	0·07^b^
Total primary bile acids	0·65	0·09	1·02	0·05^a**^	0·92	0·08^a^	1·25	0·04^b^
ω-Muricholic aicd + Deoxycholic acid	0·70	0·21	7·43	0·69^a**^	4·35	0·50^b^	5·17	0·91^ab^
Lithocholic acid	1·95	0·21	2·68	0·32^a^	1·85	0·11^b^	2·05	0·20^ab^
Hyodeoxycholic acid	0·16	0·04	0·20	0·03	0·15	0·03	0·10	0·03
Ursodeoxycholic acid	0·16	0·03	0·26	0·03^a^	2·95	0·27^b^	1·31	0·73^b^
Total secondary bile acids	2·98	0·27	10·57	0·71**	9·30	0·68	8·62	1·36
Total bile acids	3·63	0·35	11·59	0·73**	10·22	0·68	9·87	1·34

Data are presented as the mean and sem of 5–6 mice in each group. Significant differences between values of the St and C groups at *P* < 0·01** and *P* < 0·05*. ^ab^Values without a common superscript letter are significantly different among values of the C, S and OS groups at *P* < 0·05. Other abbreviations are the same as shown in Table 1. Total primary bile acids represent the sum of cholic acid, a-muricholic acid + chenodeoxycholic acid and b-muricholic acid. Total secondary bile acids represent the sum of w-muricholic acid + deoxycholic acid, lithocholic acid, hyodeoxycholic acid and ursodeoxycholic acid.

### Effects of dietary stigmasterol or oxidised stigmasterol on the mRNA expression of enzymes and nuclear receptors involved in cholesterol synthesis and catabolism in the liver

**Figure 2.**
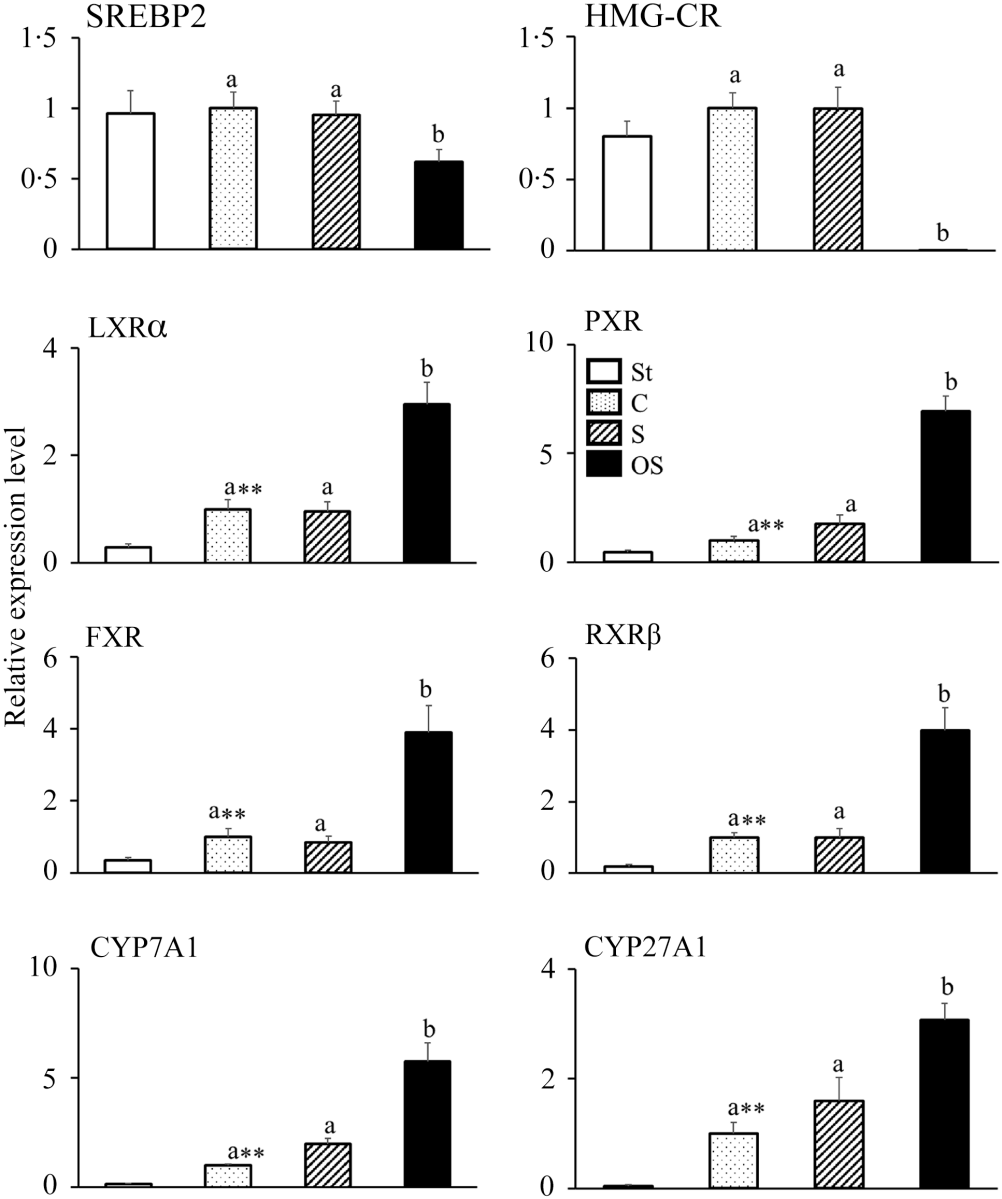
Effects of dietary stigmasterol or oxidised stigmasterol on the mRNA expression of enzymes and nuclear receptors involved in cholesterol synthesis and catabolism in the liver. Data are presented as the mean and sem of 5–6 mice in each group. Significant differences between values of the St and C groups at *P* < 0·01**. ^ab^Values without a common superscript letter are significantly different among values of the C, S and OS groups at *P* < 0·05. Other abbreviations are the same as shown in Table 1. Each relative expression level was calculated by comparing the expression level of the target gene to that of the reference *β*-actin, with the C group set to 1.

The mRNA expression levels of following nuclear receptors and enzymes involved in cholesterol synthesis did not differ between the St and C groups but were significantly lower in the OS group than in the C and S groups: SREBP2, a nuclear receptor that upregulates mRNA expression in cholesterol synthesis; HMG-CR, a rate-limiting enzyme of the cholesterol synthesis pathway in the liver (Figure 2).

In contrast, the mRNA expression levels of following nuclear receptors and enzymes involved in cholesterol catabolism were significantly higher in the C group than in the St group and significantly higher in the OS group than in the C and S groups: LXR*α*, a nuclear receptor that upregulates the mRNA expression of CYP7A1; PXR, a nuclear receptor that upregulates the mRNA expression of CYP27A1; FXR, a nuclear receptor that is activated by bile acids; RXR*β*, a nuclear receptor that forms heterodimers with LXR*α*, PXR and FXR; CYP7A1, a rate-limiting enzyme of the classic pathway of cholesterol catabolism in the liver and CYP27A1, a rate-limiting enzyme of the alternative pathway of cholesterol catabolism in the liver.

### Effects of dietary stigmasterol or oxidised stigmasterol on the mRNA expression of transporters involved in sterol absorption in the mucosa of the small intestine

**Figure 3.**
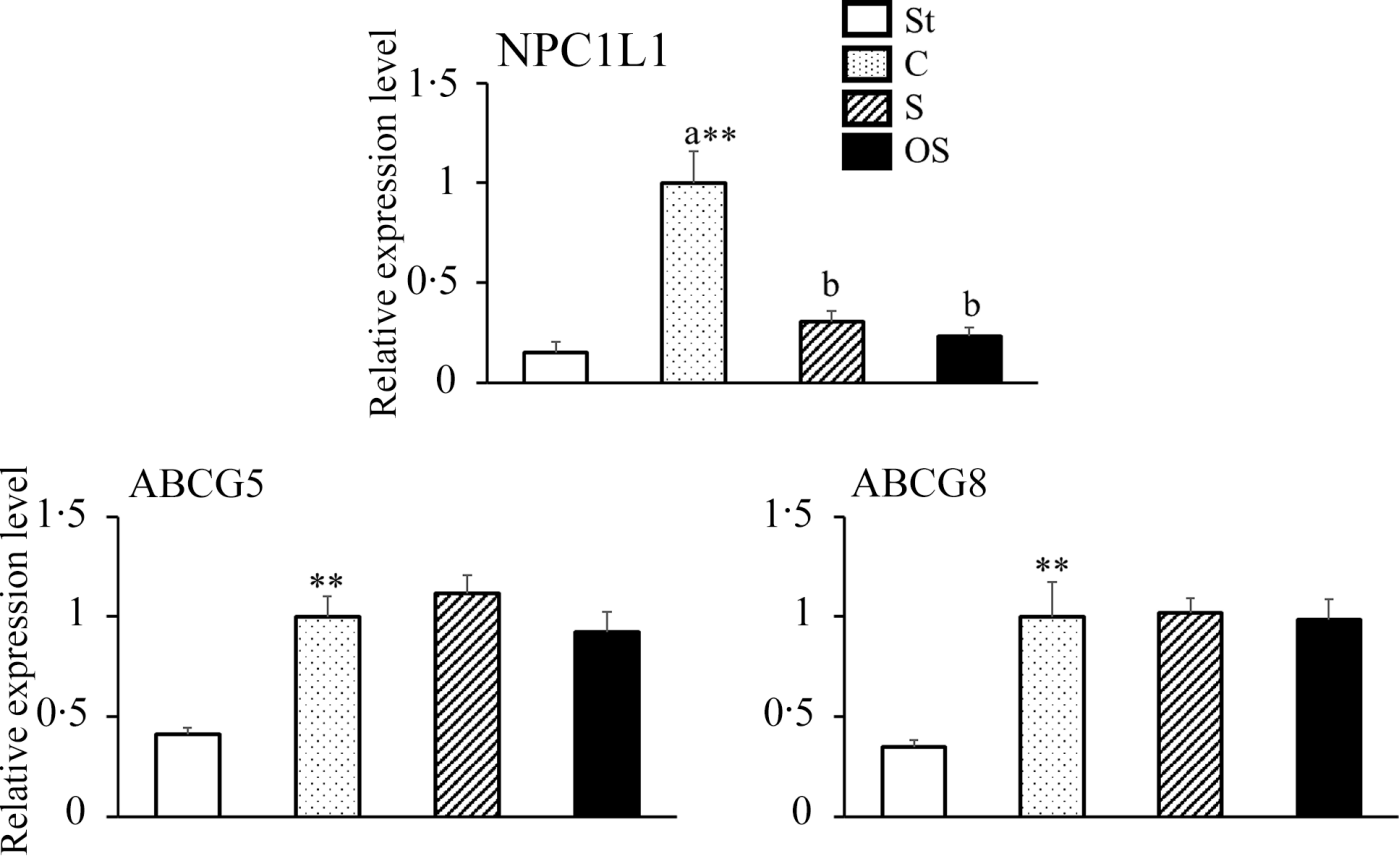
Effects of dietary stigmasterol or oxidised stigmasterol on the mRNA expression of transporters involved in sterol absorption in the mucosa of the small intestines. Data are presented as the mean and sem of 5–6 mice in each group. Significant differences between values of the St and C groups at *P* < 0·01**. ^ab^Values without a common superscript letter are significantly different among values of the C, S and OS groups at *P* < 0·05. Other abbreviations are the same as shown in Table 1. Each relative expression level was presented using the same calculation method as described in the footnote of Figure 2.

The mRNA expression level of NPC1L1, a transporter involved in cholesterol absorption in the small intestine, was significantly higher in the C group than in the St group, but significantly lower in the S and OS groups than in the C group (Figure 3). The mRNA expression levels of ABCG5 and ABCG8, transporters involved in cholesterol excretion in the small intestine, were significantly higher in the C group than in the St group; however, no significant differences were observed in the S and OS groups compared with the C group.

### Effect of stigmasterol or oxidised stigmasterol on the incorporation of cholesterol into micellar solutions

**Figure 4.**
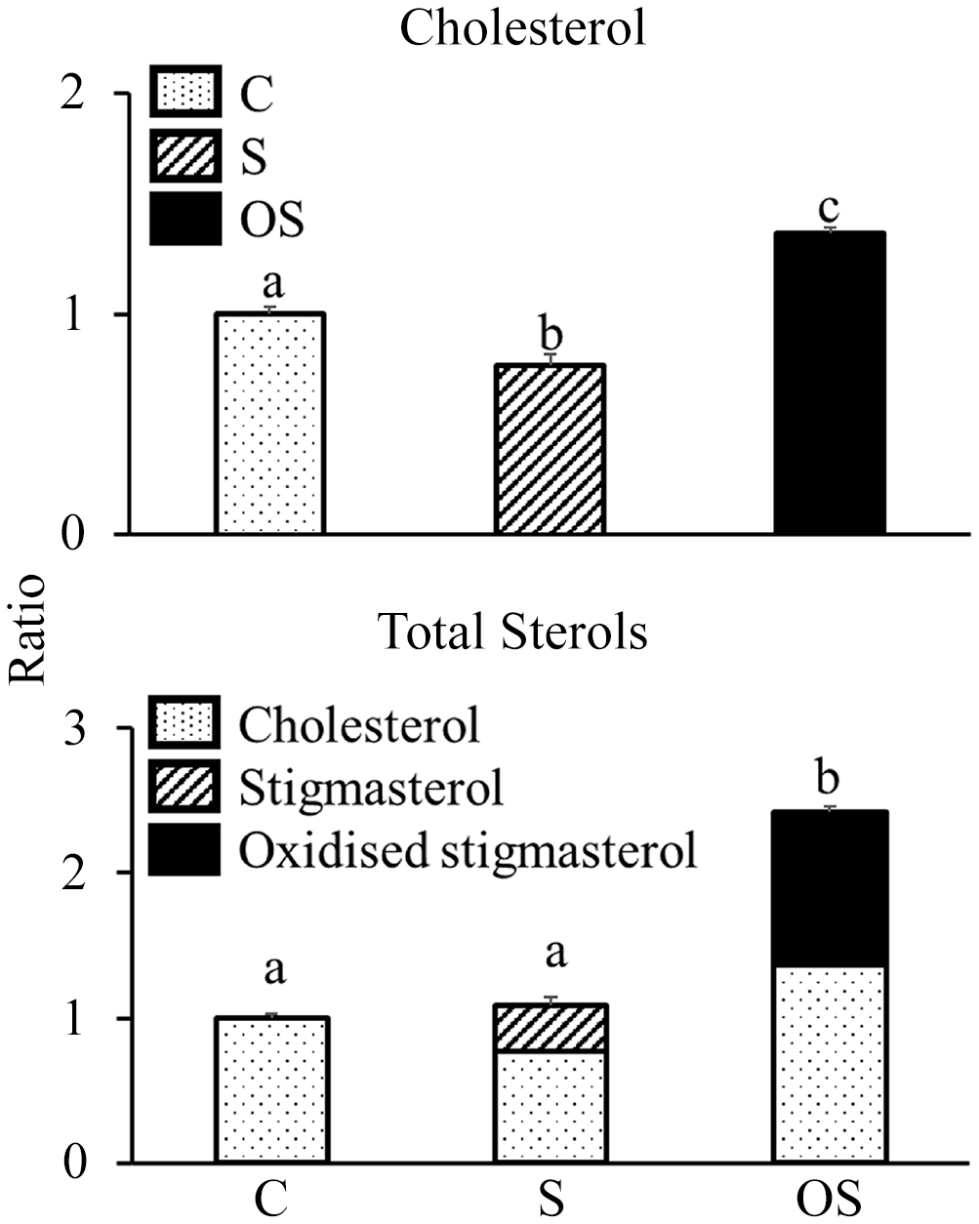
Effect of stigmasterol or oxidised stigmasterol on the incorporation of cholesterol into micellar solutions. Data are presented as the mean and sem of 4 samples in each group. ^ab^Values without a common superscript letter are significantly different among values of the C, S and OS groups at *P* < 0·05. Other abbreviations are the same as shown in Table 1.

Cholesterol levels in the micellar solutions were significantly lower in the S group than in the C group, but were significantly higher in the OS group than in the C and S groups (Figure 4). Total sterol (cholesterol + stigmasterol + OS) levels did not differ between the C and S groups but were significantly higher in the OS group than in the C and S groups. Moreover, the OS in micellar solutions of the OS group consisted of the following components: 5·5 % 4*β*-hydroxystigmasterol, 6·1 % 7*α*-hydroxystigmasterol, 12·2 % 7*β*-hydroxystigmasterol, 15·7 % 5*α*,6*α*-epoxystigmasterol, 8·7 % 5*β*,6*β*-epoxystigmasterol, 2·3 % stigmastanetriol, 0·0 % 7-ketostigmasterol and 49·5 % unidentified OS.

## Discussion

Dietary phytosterols exert hypocholesterolemic effects^(4)^ by inhibiting cholesterol absorption from the small intestine. Moreover, functional foods enriched with phytosterols may have increased levels of oxidised phytosterols during storage. Therefore, it is expected that oxidised phytosterols will be consumed at a substantial level in the daily diet. Unlike cholesterol, phytosterols are comprised of multiple compounds. Hence, the specific effects of individual oxidised phytosterols remain unclear. In this study, we examined the effects of dietary OS on cholesterol metabolism in mice, focusing on its potential harmful effects, because the effects of individual oxidised phytosterols remain unexplored *in vivo*.

We observed that dietary stigmasterol, but not OS, decreased plasma TC levels in mice. Moreover, we observed that dietary OS, unlike stigmasterol, increased cholesterol levels in micellar solutions *in vitro*. Thus, OS did not displace the cholesterol in micellar solutions. Therefore, it was expected that dietary OS would not exert hypocholesterolemic effects. Dietary stigmasterol inhibits cholesterol absorption in the small intestine by competitively inhibiting cholesterol incorporation into micelles in the intestinal lumen^(32)^. However, Liang *et al.* ^(13)^ and Wang *et al.* ^(33)^ reported that dietary oxidised phytosterols do not decrease plasma cholesterol levels. Therefore, upon oxidation, dietary stigmasterol may lose its inhibitory effects on cholesterol absorption in the small intestine.

We observed that dietary OS decreased hepatic TC levels in mice, although it did not exert hypocholesterolemic effects. This may be because of the inhibitory effect on cholesterol synthesis and promotion of cholesterol catabolism in the liver. Dietary OS decreased the mRNA expression of SREBP2 and HMG-CR in the liver. Liang *et al.* ^(13)^ also reported that dietary OS decreases the mRNA expression of HMG-CR in hamster livers. Moreover, Koyama *et al.* ^(34)^ found that dietary oxidised phytosterols decreased the mRNA expression of HMG-CR and TC in the liver. Similarly, Osada *et al.* ^(35,36)^ observed that dietary oxidised cholesterol decreased HMG-CR activity and TC levels in the liver. Thus, dietary OS may decrease hepatic TC levels by inhibiting cholesterol synthesis. In contrast, we observed that dietary OS increased faecal total primary bile acid levels and the mRNA expression levels of LXR*α*, PXR, FXR, RXR*β*, CYP7A1 and CYP27A in the liver. Future studies should clarify the relationship between the changes in individual bile acid levels and nuclear receptors such as LXR and FXR. Oxidised cholesterol acts as an LXR*α* agonist^(37)^. Plat *et al.* ^(38)^ and Kaneko *et al.* ^(39)^ showed that phytosterols act as an LXR agonist *in vitro*. Yang *et al.* ^(40)^ have reported that dietary stigmasterol acts as an LXR agonist *in vivo*. Previously, we found that dietary OS accumulates in the liver^(22)^; therefore, accumulated OS in the liver may also act as an LXR agonist, which may decrease hepatic TC levels by promoting cholesterol catabolism. Future studies should examine the effects of dietary OS on each nuclear receptor.

We observed that dietary stigmasterol and OS increased faecal cholesterol, coprostanol and neutral steroid levels. This may have occurred because dietary stigmasterol and OS suppressed the mRNA expression of NPC1L1 in the small intestine. Alrefai *et al.* ^(41)^ found that the mRNA overexpression of SREBP2 significantly increased the mRNA expression of NPC1L1; therefore, SREBP2 may upregulate the mRNA expression of NPC1L1 in Caco-2 cells. Meng *et al.* ^(42)^ reported that chlorogenic acid administration in mice suppressed the nuclear translocation of SREBP2 in the liver and downregulated the mRNA expression of NPC1L1 in the mucosa of the small intestine. Duval *et al.* ^(43)^ reported that LXR*α* is involved in the regulation of the mRNA expression of NPC1L1, and an LXR*α* agonist may downregulate the mRNA expression of NPC1L1 in mice and Caco-2/TC7 cells. Therefore, dietary stigmasterol may directly regulate the mRNA expression of NPC1L1, whereas dietary OS may reduce the mRNA expression of SREBP2 and act as an LXR*α* agonist, thus reducing the mRNA expression of NPC1L1. This study examined only mRNA expression levels. Therefore, further study is required to examine the protein levels to clarify the specific effects of OS.

In summary, dietary stigmasterol decreased hepatic TC level by inhibiting cholesterol absorption from the small intestine, whereas dietary OS may decrease hepatic TC level by decreasing cholesterol absorption from the small intestine by acting as an LXR agonist in addition to suppressing hepatic cholesterol synthesis and promoting cholesterol catabolism. Although OS has been reported to exhibit cytotoxicity^(44)^, it may be of interest as a hepatic cholesterol-lowering agent via LXR activation. Future studies should investigate the effects of OS on LXR*α* in detail.

Interestingly, we observed that the composition of the OS in micellar solutions differed from that of the prepared OS. Therefore, the incorporation of OS into micellar solutions may depend on the molecular species. In fact, 7-hydroxystigmasterols were easily incorporated into micellar solutions; however, 7-ketostigmasterol is barely incorporated into micellar solutions. This difference may be related to the differences in the composition of accumulated OS in the liver. In our previous study, we observed the accumulation of oxidised derivatives other than 7-ketostigmasterol in the liver of mice fed with OS^(22)^. Liang *et al.* ^(13)^ reported that 7-hydroxystigmasterol and 5,6-epoxystigmasterol were detected in the livers of hamsters fed 0·1 % OS, whereas 7-ketostigmasterol was not detected, despite its level being the highest among the OSs. Therefore, it is necessary to examine the relationship between the incorporation of OS into micellar solutions and its bioaccumulation.

Thus, we found that dietary OS, unlike stigmasterol, does not have an inhibitory effect on cholesterol absorption from the small intestine and modulates cholesterol metabolism in mice. In this study, a high level of OS was administered to clarify its effects over a short period, corresponding to approximately 0·34 g/kg/d in humans. However, future studies should examine the long-term feeding of each sterol at lower levels using a large number of mice to consider the effect on human health because high levels of each sterol were fed to the mice in this study. A mixture of OS derivatives was administered in this study. Therefore, it is necessary to examine the effects of individual oxidised derivatives on cholesterol metabolism *in vivo* because many of these derivatives remain unidentified.

### Conclusion

In this study, we revealed that dietary OS, unlike stigmasterol, may lose its inhibitory effect on cholesterol absorption from the small intestine because it cannot displace cholesterol in micellar solutions. However, it may decrease hepatic TC levels by inhibiting cholesterol synthesis and promoting cholesterol catabolism. Furthermore, it may inhibit cholesterol absorption from the small intestine by downregulating the mRNA expression of NPC1L1 in the small intestine.

These findings provide new insights into the biological effects of dietary oxidised phytosterols, particularly OS. While these results were obtained in mice, the mechanisms may also be relevant to cholesterol absorption and metabolism in humans. They underscore the need for further studies on the underlying molecular mechanisms. Research focusing on individual oxidised phytosterols may contribute to the development of safer functional foods and supplements enriched with phytosterols.

## References

[1] Hirai K , Shimadzu C , Takezoe R , et al. (1986) Cholesterol, phytosterol, and polyunsaturated fatty acid levels in 1982 and 1957 Japanese diets. J Nutr Sci Vitaminol 32, 363–372. 10.3177/jnsv.32.363 3806251

[2] Jaceldo SK , Lütjohann D , Sirirat R , et al. (2017) Variations in dietary intake and plasma concentrations of plant sterols across plant-based diets among North American adults. Mol Nutr Food Res 61, 1600828. 10.1002/mnfr.201600828 PMC5533645 28130879

[3] Jiménez EA , Santos HAB & Saura CF (2006) Common sources and estimated intake of plant sterols in the Spanish diet. J Agric Food Chem 54, 3462–3471. 10.1021/jf053188k 16637708

[4] Lees AM , Mok HY , Lees RS , et al. (1977) Plant sterols as cholesterol-lowering agents: clinical trials in patients with hypercholesterolemia and studies of sterol balance. Atherosclerosis 28, 325–338. 10.1016/0021-9150(77)90180-0 597345

[5] Dutta PC & Appeleqvist LA (1997) Studies on phytosterol oxides I: effects of storage on the content in potato chips prepared in different vegetable oils. J Am Oil Chem Soc 74, 647–657. 10.1007/s11746-997-0197-7

[6] Dutta PC (1997) Studies on phytosterol oxides. II: content in some vegetable oils and in French fries prepared in these oils. J Am Oil Chem Soc 74, 659–666. 10.1007/s11746-997-0198-6

[7] Menéndez CM , Ansorena D & Astiasarán I (2008) Stability of sterols in phytosterol-enriched milk under different heating conditions. J Agric Food Chem 56, 9997–10002. 10.1021/jf802000m 18928298

[8] Scholz B , Guth S , Engel KH , et al. (2015) Phytosterol oxidation products in enriched foods: occurrence, exposure, and biological effects. Mol Nutr Food Res 59, 1339–1352. 10.1002/mnfr.201400922 25787244

[9] Ryan E , Chopra J , McCarthy F , et al. (2005) Qualitative and quantitative comparison of the cytotoxic and apoptotic potential of phytosterol oxidation products with their corresponding cholesterol oxidation products. Br J Nutr 94, 443–451. 10.1079/bjn20051500 16176617

[10] Alemany L , Laparra JM , Barberá R , et al. (2013) Relative expression of cholesterol transport-related proteins and inflammation markers through the induction of 7-ketosterol-mediated stress in Caco-2 cells. Food Chem Toxicol 56, 247–253. 10.1016/j.fct.2013.02.040 23454145

[11] Plat J , Theuwissen E , Husche C , et al. (2014) Oxidised plant sterols as well as oxycholesterol increase the proportion of severe atherosclerotic lesions in female LDL receptor^+/−^ mice. Br J Nutr 111, 64–70. 10.1017/s0007114513002018 23773414

[12] Schroepfer GJ Jr (2000) Oxysterols: modulators of cholesterol metabolism and other processes. Physiol Rev 80, 361–554. 10.1152/physrev.2000.80.1.361 10617772

[13] Liang YT , Wong WT , Guan L , et al. (2011) Effect of phytosterols and their oxidation products on lipoprotein profiles and vascular function in hamster fed a high cholesterol diet. Atherosclerosis 219, 124–133. 10.1016/j.atherosclerosis.2011.06.004 21719014

[14] Laparra JM , Alfonso GA , Alegría A , et al. (2015) 7keto-stigmasterol and 7keto-cholesterol induce differential proteome changes to intestinal epitelial (Caco-2) cells. Food Chem Toxicol 84, 29–36. 10.1016/j.fct.2015.06.021 26140950

[15] Koyama T & Osada K (2025) Exogenous oxidised phytosterol may modulate linoleic acid metabolism through upregulation of fatty acid desaturase in rats. Lipids 60, 303–315. 10.1002/lipd.12444 40204289 PMC12434575

[16] Foley DA , O’Callaghan Y , O’Brien NM , et al. (2010) Synthesis and characterization of stigmasterol oxidation products. J Agric Food Chem 58, 1165–1173. 10.1021/jf9024745 20025271

[17] Batta AK , Xu G , Bollineni JS , et al. (2005) Effect of high plant sterol-enriched diet and cholesterol absorption inhibitor, SCH 58235, on plant sterol absorption and plasma concentrations in hypercholesterolemic wild-type Kyoto rats. Metabolism 54, 38–48. 10.1016/j.metabol.2004.08.004 15562378

[18] Jie F , Yang X , Yang B , et al. (2022) Stigmasterol attenuates inflammatory response of microglia via NF-κB and NLRP3 signaling by AMPK activation. Biomed Pharmacother 153, 113317. 10.1016/j.biopha.2022.113317 35772378

[19] Wang J , Huang M , Yang J , et al. (2017) Anti-diabetic activity of stigmasterol from soybean oil by targeting the GLUT4 glucose transporter. Food Nutr Res 61, 1364117. 10.1080/16546628.2017.1364117 28970778 PMC5614214

[20] Wang W-L , Chen S-M , Lee Y-C , et al. (2022) Stigmasterol inhibits cancer stem cell activity in endometrial cancer by repressing IGF1R/mTOR/AKT pathway. J Funct Foods 99, 105338. 10.1016/j.jff.2022.105338

[21] Tomoyori H , Kawata Y , Higuchi T , et al. (2004) Phytosterol oxidation products are absorbed in the intestinal lymphatics in rats but do not accelerate atherosclerosis in apolipoprotein E-deficient mice. J Nutr 134, 1690–1696. 10.1093/jn/134.7.1690 15226455

[22] Ohara Y & Osada K (2024) Effect of dietary oxidised stigmasterol on the antioxidant system in mice. J Oleo Sci 73, 1493–1503. 10.5650/jos.ess24167 39617431

[23] Menéndez CM , García HC , Astiasarán I , et al. (2008) Validation of a gas chromatography–mass spectrometry method for the analysis of sterol oxidation products in serum. J Chromatogr B Analyt Technol Biomed Life Sci 864, 61–68. 10.1016/j.jchromb.2008.01.036 18272439

[24] Conchillo A , Cercaci L , Ansorena D , et al. (2005) Levels of phytosterol oxides in enriched and nonenriched spreads: application of a thin-layer chromatography−gas chromatography methodology. J Agric Food Chem 53, 7844–7850. 10.1021/jf050539m 16190640

[25] Leal CEJ , Inchingolo R , Cardenia V , et al. (2015) Effect of microwave heating on phytosterol oxidation. J Agric Food Chem 63, 5539–5547. 10.1021/acs.jafc.5b00961 25973984

[26] American Institute of Nutrition (1977) Report of the American Institute of Nutrition ad hoc Committee on Standards for Nutritional Studies. J Nutr 107, 1340–1348. 10.1093/jn/107.7.1340 874577

[27] Folch J , Ascoli I , Lees M , et al. (1951) Preparation of lipide extracts from brain tissue. J Biol Chem 191, 833–841. 14861228

[28] Sperry WM & Webb M (1950) A revision of the schoenheimer-sperry method for cholesterol determination. J Biol Chem 187, 97–106. 14794694

[29] Isaksson G , Asp NG & Ihse I (1983) Effects of dietary fiber on pancreatic enzyme activities of ileostomy evacuates and on excretion of fat and nitrogen in the rat. Scand J Gastroenterol 18, 417–423. 10.3109/00365528309181617 6200923

[30] Sugano M , Yamada Y , Yoshida K , et al. (1988) The hypocholesterolemic action of the undigested fraction of soybean protein in rats. Atherosclerosis 72, 115–122. 10.1016/0021-9150(88)90071-8 3063266

[31] Ogino Y , Osada K , Nakamura S , et al. (2007) Absorption of dietary cholesterol oxidation products and their downstream metabolic effects are reduced by dietary apple polyphenols. Lipids 42, 151–161. 10.1007/s11745-006-3008-2 17393221

[32] Katan MB , Grundy SM , Jones P , et al. (2003) Efficacy and safety of plant stanols and sterols in the management of blood cholesterol levels. Mayo Clin Proc 78, 965–978. 10.4065/78.8.965 12911045

[33] Wang M , Yang B , Shao P , et al. (2021) Sterols and sterol oxidation products: effect of dietary intake on tissue distribution in ApoE-deficient mice. J Agric Food Chem 69, 11867–11877. 10.1021/acs.jafc.1c03648 34586790

[34] Koyama T , Fukuoka D & Osada K (2024) Effects of dietary oxidised phytosterol on lipid metabolism in rats. J Oleo Sci 73, 1189–1199. 10.5650/jos.ess24064 39168626

[35] Osada K , Kodama T , Cui L , et al. (1994) Effects of dietary oxidised cholesterol on lipid metabolism in differently aged rats. Biosci Biotechnol Biochem 58, 1062–1069. 10.1271/bbb.58.1062

[36] Osada K , Kodama T , Noda S , et al. (1995) Oxidised cholesterol modulates age-related change in lipid metabolism in rats. Lipids 30, 405–413. 10.1007/bf02536298 7637560

[37] Schwartz K , Lawn RM & Wade DP (2000) ABC1 gene expression and ApoA-I-mediated cholesterol efflux are regulated by LXR. Biochem Biophys Res Commun 274, 794–802. 10.1006/bbrc.2000.3243 10924356

[38] Plat J , Nichols JA & Mensink RP (2005) Plant sterols and stanols: effects on mixed micellar composition and LXR (target gene) activation. J Lipid Res 46, 2468–2476. 10.1194/jlr.m500272-jlr200 16150823

[39] Kaneko E , Matsuda M , Yamada Y , et al. (2003) Induction of intestinal ATP-binding cassette transporters by a phytosterol-derived liver X receptor agonist. J Biol Chem 278, 36091–36098. 10.1074/jbc.m304153200 12847102

[40] Yang C , Yu L , Li W , et al. (2004) Disruption of cholesterol homeostasis by plant sterols. J Clin Invest 114, 813–822. 10.1172/jci22186 15372105 PMC516266

[41] Alrefai WA , Annaba F , Sarwar Z , et al. (2007) Modulation of human Niemann-Pick C1-like 1 gene expression by sterol: role of sterol regulatory element binding protein 2. Am J Physiol Gastrointest Liver Physiol 292, G369–376. 10.1152/ajpgi.00306.2006 17008555

[42] Meng C , Zhou L , Huang L , et al. (2024) Chlorogenic acid regulates the expression of NPC1L1 and HMGCR through PXR and SREBP2 signaling pathways and their interactions with HSP90 to maintain cholesterol homeostasis. Phytomedicine 123, 155271. 10.1016/j.phymed.2023.155271 38103317

[43] Duval C , Touche V , Tailleux A , et al. (2006) Niemann-Pick C1 like 1 gene expression is down-regulated by LXR activators in the intestine. Biochem Biophys Res Commun 340, 1259–1263. 10.1016/j.bbrc.2005.12.137 16414355

[44] O’Callaghan YC , Foley DA , O’Connell NM , et al. (2010) Cytotoxic and apoptotic effects of the oxidized derivatives of stigmasterol in the U937 human monocytic cell line. J Agric Food Chem 58, 10793–10798. 10.1021/jf1023017 20828195

